# Glossary

**Published:** 2003

Acetaldehyde: A toxic product that results from the breakdown of alcohol by the enzyme *alcohol dehydrogenase*.
Adenosine triphosphate (ATP): A molecule, generated largely in the *mitochondria*, that provides the energy needed for many key *metabolic* reactions.
Alcohol dehydrogenase (ADH): An *enzyme* that breaks down alcohol by *oxidation*, converting it to *acetaldehyde. (*See *cytochrome P450*.)
ALD: Alcoholic liver disease.
Amino acids: The building blocks of *proteins*. Twenty different amino acids are found in human proteins; examples include lysine and methionine.
Antibody: A *protein* produced by certain immune cells that recognizes and binds to foreign proteins, leading to the destruction of those proteins.
Antioxidant: A substance such as *glutathione* and vitamins A and E or an *enzyme* that inhibits *oxidation*, serving as a defense against harmful *free radicals*.
Apoptosis: Cell death in which the affected cell participates by activating a cascade of biochemical reactions that lead to death. (See *necrosis*.)
Ascites: Accumulation of fluid in the abdomen, one of the most common complications of advanced liver disease. The presence of ascites generally indicates a poor prognosis and high likelihood of death.
Central vein: Blood exits each liver *lobule* by way of the central vein, which feeds into the *hepatic vein*.
Cirrhosis: The most advanced form of liver disease, characterized by extensive scarring that stiffens blood vessels and distorts the internal structure of the liver, severely impairing its function. Although alcoholic cirrhosis often is progressive and fatal, it may stabilize with abstinence.
Collagen: The major *protein* of fibrous connective tissue (e.g., tendons and ligaments) involved in the production of scar tissue; produced in the liver by *stellate cells*.
Cytochrome P450: A family of *cytochromes*, one of which (CYP2E1) can oxidize alcohol to form *acetaldehyde*. Most alcohol taken into the body is oxidized by *alcohol dehydrogenase;* high alcohol levels stimulate CYP2E1 activity.
Cytochromes: Specialized *enzymes* within *mitochondria* and other cell structures. Different cytochromes play important roles in *metabolizing* toxic substances, drugs, and other chemicals, as well as in producing *adenosine triphosphate (ATP)*.
Cytokines: A family of molecules, produced primarily by cells of the immune system, which regulate cellular interactions and other functions. Many cytokines play important roles in initiating and regulating *inflammation*.
Cytoskeleton: The filaments and microtubules that serve to give the cell shape and coherence.
Cytosol: Fluid contained within the cell, where several biochemical reactions (e.g., glycolysis) take place.
DNA: A family of large molecules within the cells of an organism that carry genetic information specifying the structure of *proteins*.
Endotoxin: A highly toxic chemical component of the cell walls of bacteria that occur normally in the intestine. Endotoxin can be released into the bloodstream when the bacteria die.
Endocytosis: Mechanism by which specific molecules are ingested into the cell.
Endoplasmic reticulum: A system of folded membranes that loop back and forth, spreading throughout the cytoplasm and providing a large surface area for cell reactions.
Enzyme: A substance, usually a *protein*, that directs and accelerates chemical reactions in the body but does not itself undergo permanent change.
Extracellular matrix: The body substance within which tissue cells are embedded.
Fatty acids: A building block of fat molecules. Alcohol interferes with the normal *metabolism* of fatty acids and promotes the deposit of dietary fat in the liver.
Fibrosis: The formation of scar tissue.
Free radicals: Highly reactive molecular fragments that frequently contain oxygen. (See *reactive oxygen species*.)
Glutathione (GSH): An *antioxidant* molecule found naturally in the body, composed of three *amino acids* (i.e., glutamate, cysteine, and glycine).
Hepatic encephalopathy: A potentially fatal brain disorder that results when prolonged liver dysfunction caused by excessive alcohol consumption leads to the accumulation of toxic substances in the brain.
Hepatic vein: A large vessel that receives blood after it has passed through the *central veins* of the liver *lobules*.
Hepatitis: Generalized *inflammation* of the liver, often accompanied by tissue death and *fibrosis*. Alcoholic hepatitis can be fatal, but may be reversible with abstinence.
Hepatocytes: The principal cells of the liver, which carry out most of the liver’s *metabolic* activities.
Hypoxia: Lower-than-normal levels of oxygen.
Inflammation: A defensive response to local tissue injury or infection, serving to prevent the spread of injury and activate the immune system; regulated by *cytokines*. Prolonged or excessive *inflammation* can damage healthy tissue, as in *alcoholic liver disease*.
Interferons: A group of *proteins* that increase the resistance of cells to viral infection. Interferons also act as *cytokines* and can enhance some immune responses.
Interleukins: *Cytokines* of the immune system.
Intragastric infusion model: A method of rigorously controlling animals’ consumption of alcohol and dietary nutrients by feeding a liquid diet through a tube permanently inserted in the stomach. Used with rats and mice, this model also allows researchers to monitor alcohol intake daily without having to obtain blood.
Kupffer cells: Specialized immune cells in the liver that filter bacteria and other foreign substances from the blood and produce *antibodies* and *cytokines*. (See also *sinusoids*.)
Lipid peroxidation: The sequential breakdown of fatty substances in cells by chemical *oxidation*, leading eventually to the destruction of membranes within and surrounding the cell.
Lipids: Fatty substances, including simple fats, their major components (i.e., *fatty acids*), and various fat-soluble substances (e.g., cholesterol).
Lobule: A cylindrical structure about 2 millimeters in diameter, the lobule is the basic functional unit of the liver. The liver can be composed of up to 100,000 lobules.
Macrophage: A *phagocyte* residing in tissues throughout the body. In addition to ingesting foreign particles and microorganisms, macrophages synthesize *proteins* and other substances important in *inflammatory* responses, including *cytokines*. Macrophages that reside in the liver are called *Kupffer cells*.
Metabolism: The totality of chemical reactions occurring in a cell, an organ, or the body. The term sometimes is applied more narrowly to the breakdown of a particular substance (e.g., alcohol) by specific *enzymes*.
Microsomal ethanol-oxidizing system (MEOS): An *enzyme* system that breaks down alcohol and generates toxic products such as *acetaldehyde* and oxygen radicals.
Mitochondria: Structures within cells that generate most of the cells’ energy through the production of *adenosine triphosphate (ATP)*.
NAD/NADH: Nicotinamide adenine dinucleotide (NAD) is a molecule that binds with hydrogen atoms during alcohol *metabolism* and becomes reduced NAD, or NADH. NAD and NADH move hydrogen atoms back and forth between various *oxidation–reduction* reactions, helping to maintain balance between *oxidation* and *reduction* in the cell.
Necrosis: Cell death that occurs in response to adverse conditions in the cell’s environment. (See *apoptosis*.)
Oxidation: A chemical reaction that usually involves removing a hydrogen atom from a molecule or adding oxygen to it, or both. (See *reduction*.)
Oxidative stress: An imbalance between *oxidants* (e.g., *free radicals*) and *antioxidants* that can lead to excessive *oxidation* and cell damage.
Periportal: Referring to the region of a liver *lobule* adjacent to a branch of the *portal vein*.
Perivenous: Referring to the region of a liver *lobule* surrounding a branch of the *hepatic vein*.
Phagocyte: A white blood cell capable of ingesting foreign particles and microorganisms. Phagocytes include monocytes, *macrophages*, and neutrophils.
Plasma membrane: The outer membrane of a cell. Also called the cell membrane or cell wall.
Portal vein: A large blood vessel that carries dissolved nutrients from the intestine directly to the liver.
Proteinase: A type of *enzyme* that catalyzes the breakdown of *proteins*. (See *proteolysis*.)
Proteins: Large molecules composed of long chains of *amino acids* linked together. Proteins help maintain the cell’s structure and participate in many biological functions, including the regulation of *metabolic* reactions. The shape and function of a protein is determined by the sequence of its *amino acids*.
Proteolysis: The breakdown, or degradation, of *proteins* into their building blocks, the *amino acids*. Proteolysis is essential for cell survival because some proteins must be broken down in order to carry out their biological functions, and because resulting amino acids are converted into energy molecules or recycled to produce new proteins. (See *proteinase*.)
Reactive oxygen species (ROS): Highly reactive oxygen-containing *free radicals* that are generated during *oxidative metabolism*. ROS can react with and damage *lipids, proteins*, and *DNA* in cells, causing *oxidative stress*. Common ROS include hydrogen peroxide, *superoxide* radicals, and hydroxyl radicals.
Receptor: A *protein* on the surface of a cell that recognizes and binds to chemical messengers.
Reduction: The reverse of *oxidation*, reduction is a chemical reaction that usually involves removing an oxygen atom from a molecule, or adding hydrogen to it, or both.
Respiratory chain: The electron transport system located in the mitochondria, in which electrons released by NADH are passed on to a series of other molecules that first accept the electrons and then pass them on to the next molecule in the chain. Finally. the electrons are transferred to oxygen to generate water. These successive reactions provide enough energy to drive the synthesis of *ATP* molecules.
Sinusoids: Channels in a liver *lobule* that conduct blood and nutrients to the *hepatocytes*, similar to capillaries in other organs. Sinusoids are lined with *Kupffer cells*.
Stellate cell: A star-shaped liver cell that serves as the primary storage site for vitamin A compounds and fat molecules; activation of stellate cells plays a central role in the development of *fibrosis*.
Superoxide: A destructive *reactive oxygen species* produced as a byproduct of some *oxidation* reactions.
Tumor necrosis factor alpha (TNF–α): A type of *cytokine* that promotes *inflammatory* responses, stimulates neutrophils and *macrophages*, induces fever, and induces *macrophages* to produce *cytokines*.

